# A Vibrotactile Belt for Measuring Vibrotactile Acuities on the Human Torso Using Coin Motors

**DOI:** 10.3390/mi15111341

**Published:** 2024-10-31

**Authors:** Shaoyi Wang, Wei Dai, Lichao Yu, Yong Liu, Yidong Yang, Ruomi Guo, Yuemin Hong, Jianning Chen, Shangxiong Lin, Xingxing Ruan, Qiangqiang Ouyang, Xiaoying Wang

**Affiliations:** 1 Third Affiliated Hospital of Sun Yat-sen University, Sun Yat-sen University, Guangzhou 510630, China; wangsy3722@126.com (S.W.); yangyd6@mail.sysu.edu.cn (Y.Y.); chjning@mail.sysu.edu.cn (J.C.);; 2 Ultrasound Department, Guangdong Provincial Occupational Disease Prevention Hospital, Guangzhou 510300, China; 7884569@163.com; 3 College of Electronic Engineering, South China Agricultural University, Guangzhou 510642, China; 4 Guangdong Key Laboratory of Blockchain Security, Guangzhou University, Guangzhou 510006, China

**Keywords:** vibrotactile acuities, psychophysics, coin motor, vibrotactile display, human torso

## Abstract

Accurate measurement of the vibrotactile acuities of the human torso is the key to designing effective torso-worn vibrotactile displays for healthcare applications such as navigation aids for visually impaired persons. Although efforts have been made to measure vibrotactile acuities, there remains a lack of systematic studies addressing the spatial, temporal, and intensity-related aspects of vibrotactile sensitivity on the human torso. In this work, a torso-worn vibrotactile belt consisting of two crossed coin motor arrays was designed and a psychophysical study was carried out to measure the spatial, temporal, and intensity-related vibrotactile acuities of a set of human subjects wearing the designed belt. The objective parameters of vibrational intensity and the timing latency of the coin motor were also determined before measuring the vibrotactile acuities. The experimental results indicated that the tested coin motor was able to generate a median number of five and six available just-noticeable differences in intensity and duration, respectively. Among the four parameters of vibrational intensity, the perceived intensity was the most relevant to vibrational displacement. The spatial acuities measured as the degree of two-point spatial thresholds (TPTs) showed less individual difference than the distance TPTs. The results from the current work provide valuable guidance for the design of a comfortable torso-worn vibrotactile display using coin motors.

## 1. Introduction

The activation of the skin has been shown to be more effective and intuitive in cueing information than visual channels in certain cases, such as scenarios with decreased visual perception, increased noise, and the potential for disorientation, or in the presence of gravitoinertial acceleration. Employing a tactile channel may relieve other heavily loaded sensory channels, potentially providing a major safety enhancement. Vibrotactile (vibratory tactile) stimuli tend to adapt slowly, and thus, they do not need to be repeated in order to produce a sensation that will remain in conscious awareness [1]. Due to the convenience and real-time response allowing for dynamic patterns of stimulation, vibrotactile stimuli have almost universally been adopted to cue information. The continued miniaturization of vibrational motors driven by advances in mobile technology and the easily adjustable vibrotactile intensity of these motors, further promote their widespread use. There has been an increased interest in vibrotactile displays in the forms of belts [2], vests [3], and other types [4,5]. In recent years, the favorable effects of vibrotactile displays on navigational performance, situational awareness, and workload reduction have been precedingly shown for the other emerging specialties such as for the visually impaired [6], pilots [7,8], and vehicular drivers [9].

The cruciality of normally functional vibrotactile displays is the bandwidth of the information that can be transmitted [10]. In vibrotactile displays, three basic vibrational parameters are typically used for coding information: timing, intensity, and spatial location. The resolution of cueing information using the three parameters is related to the temporal, intensity-related, and spatial acuity of the skin, respectively. The skin covering the torso contains hundreds of mechanoreceptors [11], and it is capable of precisely encoding information. However, even the most advanced displays have not realized the full potential of the processing capacities of the torso [12]. The vibrotactile display in this study was easily interpolated between the surface of the torso and the coat without attracting public attention. Vibrotactile acuity has been identified as being significantly different between the four cardinal sites of the torso (the center of the left waist, center of the right waist, center of the abdomen, and center of the back) [12,13]. These four sites have been shown to perform better in localizing the vibration stimulus compared to other areas of the torso, and they have been widely used to arrange vibrotactile actuators for cueing directional information [6,7]. Therefore, the data of vibrotactile acuities on the four cardinal sites of the torso are valuable and may describe the acuities of the whole torso.

Generally, three types of electromechanical actuators are used in vibrotactile displays [10], including an eccentric rotating mass actuator, a linear resonant actuator, and piezoelectric ceramics. The eccentric rotating mass actuator generates vibrations through the centripetal force generated by an offset rotating mass. Its frequency and amplitude are directly related to the applied input voltage. The frequency and amplitude of vibration produced by an eccentric rotating mass actuator are usually coupled, i.e., increased or decreased concurrently rather than independently [14]. Conversely, the linear resonant actuator allows for changing the amplitude (at a resonance frequency) separately. For example, the voice coil actuator, a typical linear resonant actuator, permits the control of both the amplitude and frequency. The benefit of a separate change in frequency and amplitude is that when both of them are varied for the discrimination of vibrational signals, a greater number of levels can be distinguished [15]. Piezoelectric ceramics are another type of vibrotactile actuator whose characteristic is producing vibration with a wider range of frequency, lower noise, and more realistic tactile sensation than the former two types of actuators. However, a high voltage is needed to drive piezoelectric ceramics to activate vibration [16].

Among the above three types of electromechanical actuators, eccentric rotating mass motors are most widely utilized for their vibrotactile displays due to their low price, energy efficiency, and convenient control. For example, coin motors, typical eccentric rotating mass actuators, are small and energy-efficient. Their integration in electronic circuits is relatively straightforward. Due to their large dynamic range, small size, and low price, coin motors are beneficial for use in a vibrotactile display consisting of multiple actuator arrays [6,7]. Although the amplitude and frequency of coin motors cannot be controlled independently, the perceived intensity of a coin motor can be easily controlled by changing the input voltage using pulse width modulation. Compared to other actuators, a coin motor is smaller in size, cheaper, and energy-efficient [17]. Thus, it serves as an ideal alternative choice for the actuators in vibrotactile suits due to its mentioned conveniences. Measuring the vibrotactile acuities of the torso using coin motors is necessary and valuable for designing vibrotactile displays using coin motors. For example, Yang et al. previously designed a vibrotactile pedestal with multiple actuators to intuitively display information such as the position and direction for smartphone applications [18]. These types of vibrotactile suits consist of a large number of vibrating actuators. In recent years, there have been some innovative suits, such as Teslasuit designed by Tesla Studios and skinterface designed by a developer from the London Royal College of Art. Teslasuit, equipped with Electrostatic Vibration actuators, is a full-body haptic device for gaming and Virtual Reality [19]. Skinterface is a skin suit equipped with sophisticated vibration actuators which convey subtle sensations, effectively converting a virtual interaction into physical feeling [20]. Further study of the vibrotactile acuities to the vibration of an eccentric rotating mass motor on a torso will promote the vibrotactile suit’s utilization in practical applications. Overall, the coin motor was chosen as the actuator due to its compact size, energy efficiency, and ease of integration into wearable systems. Its capability to generate sufficient vibrotactile feedback makes it suitable for our application in vibrotactile displays.

In recent decades, vibrotactile thresholds have been studied by psychophysical researchers in terms of spatial length or location [12], vibration intensity [21,22], and temporal duration [12,23] using linear resonant actuator motors. Vibrotactile acuities have been measured on various skin areas such as the arm, abdomen, and fingertip [1,24,25,26]. Although previous studies of vibrotactile acuities have been fruitful, the psychophysical limits of vibrotactile acuities to three basic vibration parameters on the torso using coin motors have rarely been investigated. The psychometric equivalent of the number of perceivable acuities is the number of just-noticeable differences (JNDs). The number of available just-noticeable differences has not yet been documented for coin motors in the field of vibrotactile displays [27]. Two-point spatial thresholds (TPTs) and the temporal duration threshold are also important parameters for designing simplified and cost effective vibrotactile displays, as well as tempo-spatial vibrotactile coding. However, we are not aware of any studies assessing temporal duration JNDs and TPTs on the four cardinal sites of the torso using a coin eccentric rotating mass motor and TPTs on the back of the torso [10,28]. Studies about these were only focused on the available number of intensity JNDs on the back of torso. The available number of just-noticeable differences for temporal durations, and TPTs in other cardinal areas of the torso, as well as the relationship between the four objective vibration parameters (frequency, displacement, velocity, and acceleration) and perceived intensity, have not been systematically investigated.

**Figure 1:**
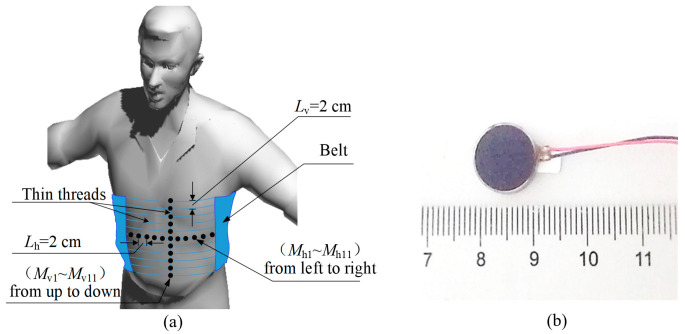
Design of the vibrotactile belt. (**a**) Concept of for the vibrotactile belt for measuring vibrotactile acuities on human torsos using two crossed tactor (black, filled circles) arrays. *L_h_* (2 cm) and *L_v_* (2 cm) represent the distances between adjacent actuators in horizontal or vertical directions. (**b**) Prototype of the flat coin motor used as a tactor in (**a**).

The goal of the current study is to measure the vibrotactile acuities in terms of temporal, spatial, and intensity acuities on the torso using coin motors. In the current study, we designed a vibrotactile belt as illustrated in Figure 1a to measure the subjective magnitude of intensity and temporal duration perception across the dynamic ranges of coin motors, and TPTs in horizontal and vertical directions of the torso using standard psychophysical procedures. Considering that people differ in waist size, the spatial acuities in the horizontal direction of the torso was measured as a scale of degree. The coupled characteristic of the coin motor may result in unpredictable perceptions in psychophysical tests, which could restrict their use in a vibrotactile display [28]. To avoid this uncertainty in tests, the objective electromechanical characteristics of the coin motor should be determined before measuring the vibrotactile acuities. In summary, this research contributes to an ongoing effort to develop a comfortable torso-worn vibrotactile display.

## 2. Measurement of Objective Parameters of the Coin Motor

The vibrotactile perception of a coin motor is strongly related to its vibration characteristics. It is necessary to measure the objective characteristics of the actuator prior to measuring the acuities of vibrotactile perception. In the following section, we will build a platform to measure the objective characteristics of the coin motor used in the current study.

Generally, an indirect parameter like driving voltage is used to represent the perceived intensity of a coin motor. However, different types of coin motors have different driving voltage ranges. In order to expand the applications of the experimental results and the facilitate comparisons with previous studies, it was necessary to measure the objective parameters (e.g., frequency or displacement) of the vibration actuators. The relationship between vibration displacement (*s_p_*, peak value), velocity (*v_p_*, peak value), are acceleration (*a_p_*, effective value) is illustrated as follows.

(1) $$\left\{\begin{array}{c} a_{p}=\frac{F_{N}}{m}\\ v_{p}=\frac{\sqrt{2}{\cdot a}_{p}}{2\cdot \pi \cdot f_{t}}\\ s_{p}=\frac{{\sqrt{2}\cdot v}_{p}}{2\pi \cdot f_{t}}\; \end{array}\right.$$

where the *F_N_* is inertial force of the mass block in the vibration meter, *m* is the mass block in the coin motor, *f_t_* is the vibration frequency. As seen from the equation, all the parameters of the motor will be determined by measuring the vibration frequency and *F_N_*.

### 2.1. Testing Platform

In the current study, we selected a flat coin motor (C1234B016F, Dongguan, China) with diameter of 12 mm (see Figure 1b) as a vibration tactor. Its rated voltage and rated rotation speed are 3 V and 9000 rpm, respectively (https://www.kotl.com.cn 21 August 2024). The intensity of vibration was controlled by the pulse width modulation (PWM) duty cycle (stimulus level) at a certain frequency. The PWM duty cycle adjusts the voltage, which in turn modifies the motor’s rotational speed and results in changes in vibration amplitude due to the unbalanced rotating mass.

**Figure 2:**
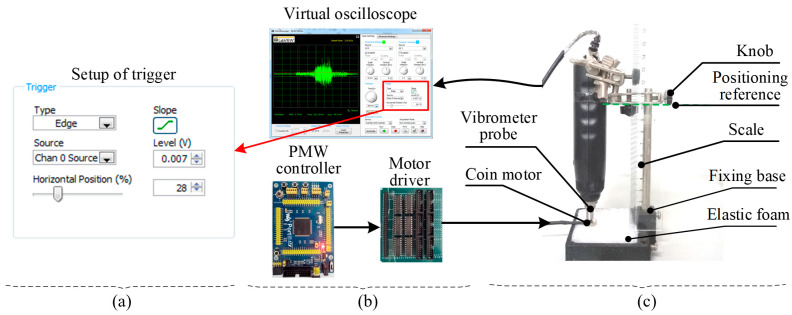
Measurement system for measuring objective parameters. (**a**) Motor controller and signal acquisition. (**b**) Mechanic platform for measurement. (**c**) Trigger setting interface for measuring latency of motor.

The system for measuring vibration amplitude (e.g., vibration displacement), frequency, and latency for the coin motor used in the current study is shown in Figure 2. The piezoelectric signal detected by an AR63B vibrometer probe was connected to a NI myDAQ digital oscilloscope through a lead wire. The AR63B vibrometer, a piezoelectric device, was used to measure the vibration force exerted by the coin motor. While the vibration amplitude (displacement) could be inferred indirectly, the measurement here focused on force. The tested motors were embedded in a piece of elastic foam. The elastic foam, which had an elasticity coefficient is approximately equal to that of skin, was selected to simulate cutaneous tissue. The knob on the slide bar can be moved to adjust the press depth (*P_d_*) of the motor which was inserted into the foam, aided with a scale measuring the press depth. The driving voltage of the motor was changed via adjusting the duty cycle of the pulse width modulation signal produced by a control board integrated with a STM32FL03ZET6 controller (Shenzhen, China). The frequency of pulse width modulation was set to about 3 kHz for a wide adjustable range of intensities and ensured stable vibration control over a wide range of intensities. By recording a series of voltage inputs, the vibration duration, peak acceleration, and peak vibration frequency were determined using fast Fourier transform (FFT) analysis.

**Figure 3:**
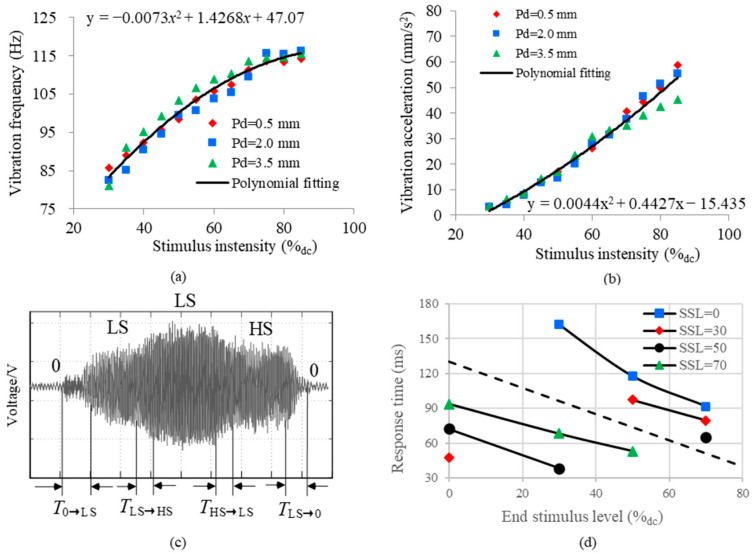
Results of objective tests of the coin motor (**a**,**b**). Average vibration frequency and displacement changing with driving voltage under different press depths. Each solid line represents a fitting curve of average data over different depths; the fitting equation was presented on the bottom-right of the figure. 100%_dc_ corresponds to 3 V. (**c**) Pressure signal curve recordings of 30 → 50 → 70 → 50 → 0 (%_dc_) for measuring the latency of the coin motor. (**d**) Curve of response time was calculated from (**c**) and it changes as a function of the end stimulus level under different SSLs.

The latency which includes onset time, shift time, and stop time is an important parameter for a vibrotactile actuator. Latency measurements focused on the on-and-off time constants, as these provide a more reliable measure of the motor’s transient response. Onset time is the time taken for the actuator to vibrate from a stationary state (i.e., the Start stimulus level (SSL) = 0%_dc_) to a stable vibrating state. The percentage (%_dc_) mentioned refers to the duty cycle of pulse width modulation, with 100% corresponding to the maximum voltage of 3V. Shift time is the time taken for the actuator to vibrate from a vibrating state to another vibrating state. The stop time is the time taken for the actuator to vibrate from a vibrating state to a stationary state. The output signal from the piezoelectric sensor in the vibrometer probe was measured in the virtual oscilloscope which was set into a waveform-capturing mode triggered by the rising edge (see Figure 3c). The motor is controlled from a low-stimulus (LS) level to a high-stimulus (HS) level then back to the LS level (i.e., LS → HS → LS) using PC software (NI LabView 2021). Considering the convenience of measuring latency, the step for changing the stimulus level is set up to 20%_d_. Thus, the intensity can be classified into four levels: 0, 30, 50 and 70%_dc_. All types of response times for combinations between two of the four stimulus levels only need measurement for four times (i.e., 30 → 50 → 30, 50 → 70 → 50, 30 → 70 → 30, and 0 → 70 → 0%_dc_). We can read the latency shifting from one voltage level to another level as illustrated in Figure 3c.

### 2.2. Results of Objective Testing

The measured characteristic of vibration frequency and the vibration amplitude with the change in driving voltage, respectively, are shown in Figure 3. As seen in Figure 3a,b, the coin motor vibrates with a frequency ranging from 80 Hz to 140 Hz, which is the perceptible frequency band of skin according to [29,30]. The vibration frequency did not change with voltage when the voltage exceeding 90%_dc_. The vibration frequency and displacement can be changed by the driving voltage but cannot be changed separately. The driving voltage should be high enough to make the vibrotactile stimulus noticeable but not exceed 90%_dc_ in order to ensure that the motor works in the non-saturated range. There was no obvious effect of the press depth on the vibration frequency and the vibration amplitude, which indicates that the vibrotactile perception is not affected by the degree of contact between the skin and the surface of the tactor.

The curve of latency as a function of end stimulus levels under different Start stimulus level (SSLs) is shown in Figure 3d. As seen in this figure, the latency decreases with end stimulus levels and SSLs, and its maximum value is not higher than 200 ms. The duration of the vibrotactile stimulus should be set to be longer than its corresponding latency. The stimulus’ rising latencies depicted in the upper part of the figure separated by the dotted line are longer than the stimulus’ falling latencies depicted in the lower part of the figure. The measured results of frequency, acceleration, and latencies will provide practical guidance for the setting of the vibration control parameters (e.g., the scale of perceptive intensity, the interstimulus interval, and the duration of the vibrotactile stimulus) in vibrotactile coding.

## 3. Psychophysical Study of Measuring Vibrotactile Acuities Using a Vibrotactile Belt

After the objective parameters of the vibration motor in the vibrotactile belt had been determined, we carried out a psychophysical study to measure the vibrotactile acuities by recruiting human subjects to wear the vibrotactile belt. The vibrotactile acuities can be determined by the perceptual threshold, which reflects the perceptual sensitivity of a sensory channel. The perceptual threshold can be classified into an absolute and a difference threshold [10]. The absolute intensity or the temporal threshold is the minimum magnitude in intensity or duration that allows a stimulus to be noticeable by human skin. The spatial two-point threshold (TPT), defined as the minimal distance necessary for recognizing a stimulus pair as two separate stimuli [10], can also be measured using vibratory stimuli. Therefore, we measured the absolute intensity threshold and the intensity just-noticeable differences (JNDs) to evaluate the intensity vibrotactile acuities, the temporal threshold and temporal JNDs to evaluate temporal acuity, and the spatial TPTs in the horizontal and vertical directions of the torso to evaluate intensity spatial acuity.

### 3.1. Experimental Setup

Fifteen subjects (10 males, 5 females) participated in this experiment. Their ages ranged from 20 to 30 years old with varied body sizes, all of whom were right-handed and reported to have no known cutaneous or kinesthetic problems. During the experiment, subjects wore earphones playing Gaussian white noise to eliminate auditory cues. For the convenience of changing the distance between two tactors for measuring spatial acuity, we designed a vibrotactile stimulus consisting of two cross-tactor arrays as shown in Figure 1a. The axes through the horizontally and vertically arranged tactors are orthogonal. In order to deduce the vibration diaphony between the adjacent actuators, we used the thin thread rather than a whole wide belt to fix the actuators. Considering the subjects’ privacy and safety, a thin underwear was worn between the belt and the naked torso. When measuring acuities on the four cardinal sites of the torso, the tactor on the intersection of the two tactor arrays was set at the center of four cardinal sites. Previous research has indicated that there is no significant difference between the two gender groups in the vibrotactile acuities [31,32].

**Figure 4:**
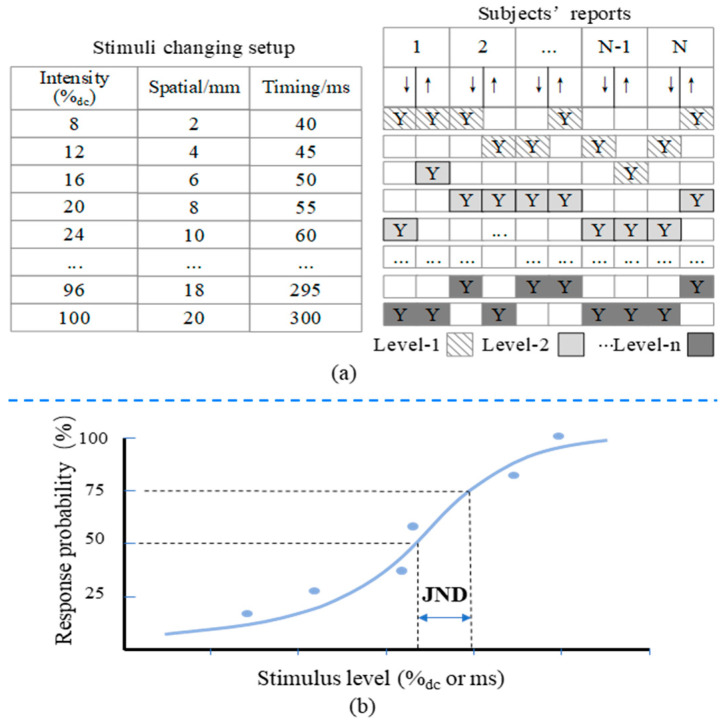
Schematic diagram of measuring vibrotactile acuity and determining a JND. (**a**) Schematic diagram of measuring vibrotactile acuity on each vibration parameter. (**b**) Example psychometric curves showing the probability of subjects perceiving one stimulus as stronger/higher than the JND.

The tactor on the intersection of the two tactor arrays was activated to measure the intensity acuity and temporal acuity. The spaces of changing intensity and timing duration are shown Figure 4. When measuring the intensity threshold, the starting intensity of the stimulus was set to the minimum level that can activate vibration. Previous vibrotactile studies indicated that the localization of the vibrotactile stimulus improves with the duration of the stimulus (DoS) ranging from 80 ms to 320 ms [33]. The subjects will feel uncomfortable if the DoS exceeds that range [34], hence, in the current study, the maximum DoS was set to 300 ms and the minimum DoS was set to the fastest response time shown in Figure 4. When measuring temporal acuities, the intensity of the vibrotactile stimulus was set to 50%_dc_. Considering that waist size varies among subjects, we take the spatial angle as the parameter to measure the spatial threshold of vibrotactile sensation in the horizontal direction of the torso. The conversion relationship between the angle threshold and the corresponding distance threshold in the horizontal direction of the torso is illustrated in the following equation.

(2) $$\theta_{ht}=\frac{L_{ht}}{W_{c}}\cdot 360$$

where *θ_ht_* (degrees) is the horizontal angle threshold and *L_ht_* is the horizontal distance threshold, respectively. *W_c_* is the waist circumference. It is better to measure the spatial threshold in the horizontal direction of the torso in the scale of degree than in distance, since people differ in size and shape. We measured the spatial threshold in the vertical direction of the torso as distance, and adopted the term *L_vt_*, which represents the spatial distance threshold in the vertical direction of the torso. When measuring the TPTs, the two tactors are activated simultaneously. Since there is no significant effect of intensity on the two-point threshold in simultaneous vibration [10], the intensity was set to 50%_dc_. The vibration duration was set to 300 ms. The spatial distance between the two activated tactors was increased from 2 cm until two discrete vibrations were obviously perceived. The actuator *M*_v1_ and *M*_h1_ were selected as the start points for the vertical and the horizontal direction, respectively.

The just-noticeable difference (JND) was defined as the amount of change in stimulus intensity necessary to detect a reliable change in sensation [35]. Using Weber’s law [36], the number of available JNDs can be determined. When the dynamic range and the JNDs at a particular reference stimulus magnitude are known, JNDs were compared via the Weber fraction:

(3) $$K=\frac{∆S}{S}=\frac{JND}{ref}$$

where *K* is the Weber fraction, Δ*S* = JNDs, and *S* is reference value used to determine the JNDs. In contrast to the JNDs which are valid only at the specific reference value used, *K* may be considered as a constant across stimulus levels (12). According Fechner’s law, the stimulus level (*S*) changes as the following function of the rating perceived magnitude (*r*):

(4) $$S={S_{th}\cdot \left(K+1\right)}^{r-1},\;r\epsilon [1,2,3,\ldots ]$$

Using the Weber fraction, the available number of JNDs in the dynamic range can be evaluated by following equation:

(5) $$N_{J}=round(\frac{ln{(S_{m}/S}_{th})}{ln(1+k)})$$

where *N_J_* = number of available JNDs, *S_m_* is the maximum comfortable stimulus level. *S_th_* is the absolute threshold of the stimulus level.

In Weber’s Law, “*S_m_”* (stimulus magnitude) refers to the magnitude of the stimulus applied in an experiment or measurement. “*K*” (the Weber constant) is a constant that expresses the proportional relationship between the change in stimulus intensity and the original stimulus intensity needed to detect the smallest noticeable difference. The just-noticeable difference—JND is directly linked to Weber’s Law, which states that the JND is a constant proportion of the original stimulus. The JND represents the minimal difference between two stimuli that can be perceived as different, as we have referred to in the previous main text. “ref” (response or reaction) refers reference stimulus refers to a baseline stimulus against which other stimuli are compared. In experiments related to Weber’s Law, the reference stimulus is used to determine the relative difference between it and the subsequent stimuli, which influences perception. “*r*” (response or reaction) refers to the subject’s response to the stimuli, particularly in relation to how the difference in stimulus intensities is perceived. These terms help quantify sensory experiences based on Weber’s Law, which demonstrates that perception changes in proportion to the intensity of the original stimulus.

### 3.2. Experimental Procedures

There are several methods for measuring the threshold of vibrotactile sensation, such as the method of limits, the method of adjustment [37], the method of constant stimuli, adaptive psychophysical methods (Staircase procedures, Bayesian, and maximum-likelihood procedures [38]), and magnitude estimation [39]. In this study, vibrotactile acuities were experimentally measured using the method of limits since it is easy to implement and convenient to measure the JNDs under different stimulus levels. The experimental procedures for measuring spatial, temporal, and intensity acuity are identical, as illustrated in Figure 4a. In the first experimental block, subjects rated a subset of 20 stimuli for practice. The practice block was followed by five experimental blocks in which archival data were collected. To reduce the error of practice and habituation that easily occurs in psychological experiments [37], the presented stimulus was changed in two directions as illustrated in Figure 4a on the right. The experimental procedure was carried with “yes/no” tasks in both increasing and decreasing order. The series of vibration stimuli with certain difference between each adjacent pair were presented in increasing and decreasing order to the subjects (see Figure 4a right). When a vibration was perceived as different from the previous one, the experimenter labeled it with a symbol “Y” as illustrated in Figure 4a on the right. The average of a level of labeled values over all subjects represents the magnitude of one scaling level, and the value of the first level is the absolute threshold. Methods for measuring vibrotactile acuity in different areas of the body and for different vibration parameters are the same. To determine the just-noticeable differences (JNDs), subjects were presented with two stimuli at different parameter settings (intensity or duration) and asked to indicate which one they perceived as stronger or longer. The probability of a correct response was recorded across multiple trials. The resulting psychometric curves were plotted for each parameter setting, and the slope of these curves was used to determine the JND as illustrated in Figure 4b. This approach follows standard procedures in psychophysics, minimizing sequence effects and improving the precision of JND measurement.

### 3.3. Experimental Results

The experimental data of vibrotactile acuities were presented in Section 3.3. We analyzed the data by employing a one-way repeated measure ANOVA. The differences were further analyzed using a post hoc Tukey test with alpha set at 0.05 [40].

**Figure 5:**
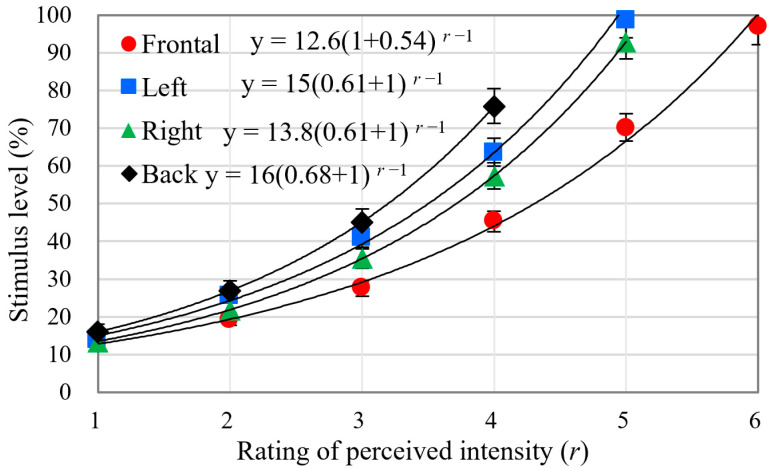
The stimulus level changes as a function of ratings of perceived intensity under different torso regions. Solid lines denote the fitting curve using Equation (4).

**Figure 6:**
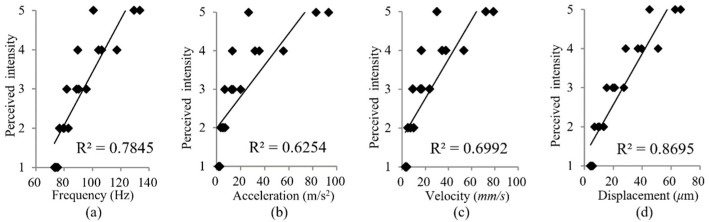
Average rating of perceived intensity changing with vibration frequency (**a**), acceleration (**b**), velocity (**c**) and displacement (**d**). Correlation coefficient (R^2^) is shown.

Intensity acuity: The distinguishable intensity levels in the regions of the torso and the corresponding stimulus levels are depicted in Figure 5. The absolute thresholds can be determined by the average stimulus value of the first perceived level (see the solid line in Figure 5). The Weber fraction of the frontal torso is lower than that of the back of the torso (see the fitting function in Figure 5), which indicates that the intensity acuity at the abdomen is higher than that at the back of the torso. There are about five, four, four, and three JNDs of intensity at the front, left, right, and back of the torso, respectively, as calculated according to Equation (5). In order to determine which of the four objective vibration parameters are most correlated with the perceived intensity, we carried out another analysis where the perceived intensity changed with different vibration parameters as shown in Figure 6. Vibration frequency and acceleration data under different set stimulus levels were calculated according to the fitting functions shown in Figure 6a,b. Thus, the data of vibration velocity and displacement were determined as referred to in Equation (1). As seen in Figure 6, the perceived intensity is best accounted for by vibration displacement (R^2^ ≈ 0.86).

**Figure 7:**
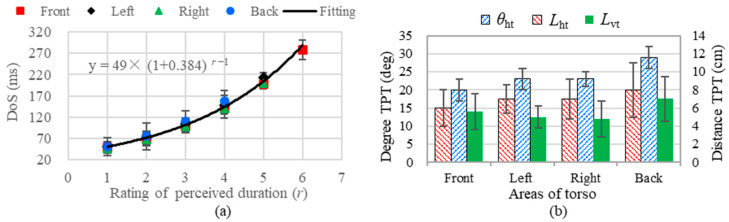
Spatial and temporal acuity of the four cardinal areas of the torso. (**a**) The DoS changes as a function of ratings of perceived duration, the solid line denotes the fitting curve of average data across the four torso areas. (**b**) Two-point spatial threshold under different torso areas.

Temporal acuity: The identifiable levels of perceiving temporal duration under different regions of the torso are illustrated in Figure 7a. DoS changes as a function of the rating of perceived duration under different torso areas. The average Weber fraction across subjects was 0.384 (see the fitting function in Figure 7a). There are six JNDs of temporal duration as calculated according to Equation (5).

Spatial acuity: The experimental results including the two-point spatial threshold of vibration under the horizontal and vertical directions are shown in Figure 7b. The degree and distance TPTs did not show significant differences between the frontal, left, and right torso (*p* > 0.5), while the degree and distance TPTs on the back of the torso are relatively higher compared to other torso areas (*p* < 0.05). The vertical spatial TPTs measured in distance are significantly higher than horizontal spatial TPTs measured in distance (*p* = 0.024). The degree threshold (average SD = 8.3%) shows less fluctuation than the distance threshold (average SD = 32.5%).

## 4. Discussion

In the current study, we measured vibrotactile acuities on the torso and examined the characteristics of coin motors. Consistent with the study by Cholewiak et al. [1], we found no significant absolute threshold differences for the temporal acuity and intensity observed across the tested torso areas. However, there was a significant difference in the spatial two-point threshold between the back of the torso and the other tested areas. The skin can discriminate between five just-noticeable differences in intensity for a coin motor. As illustrated in Figure 6, perceived intensity changes are more closely associated with vibration frequency than with acceleration, and are best accounted for by vibration displacement. Notably, the density of slowly adapting type 1 (SA1) tactile receptors on the back of the torso is lower than in other regions. Additionally, the lower back lacks Meissner’s corpuscles [41], which may influence the vibrotactile acuity we measured. As seen from Figure 5 and Figure 7a, we hypothesize that intensity and temporal acuities may be directly related due to the coin motor’s time constant, where longer excitation times may increase both motor speed and vibration intensity. This relationship should be explored in future studies.

The vibration frequency of our motor ranged from approximately 80 to 120 Hz, where the absolute intensity threshold is primarily determined by Pacinian corpuscle (PC) receptors, which are evenly distributed across glabrous skin areas [42]. Importantly, neural responses from tactile receptors are evoked by skin indentation rather than by pressing force.

Our research presents the number of identifiable levels for the sensation of intensity, which is less than that of timing, as illustrated in Figure 5 and Figure 7. Therefore, we also concluded that the human torso is more sensitive to the temporal parameters of vibration than to intensity, supporting prior findings [3]. As seen in Figure 5, the perceived intensity will not increase when the stimulus level exceeds 80%, which is in accordance with the results in Figure 3a. The number of identifiable levels of intensity and the temporal duration are strongly related to capacity of short-term memory. In accordance with the findings of prior studies [1,13,25,26], the back of torso shows the bluntest acuity spatial resolution in the tested areas. The estimations of perceived magnitude increased monotonically with increasing stimulus level (Figure 5 and Figure 7) and showed a linear logarithmic dependency on stimulus level, which is in accordance with Fechner’s law [43]. We conclude that the perceived intensity generated by coin motors across their dynamic range is associated with standard psychophysical responses.

### 4.1. Comparison with Previous Relevant Studies

Differently from the previous studies measuring vibrotactile acuities, the current study presents a method for measuring spatial, temporal, and intensity acuity simultaneously by designing a vibrotactile belt. Thus, the data of vibrotactile acuities obtained in the current study are robust. The objective parameters of a coin motor have been studied by researchers using various methods [28]. The frequency ranged from 30 to 300 Hz. The motor can generate 15 available just-noticeable differences (JNDs), capable of conveying 16 levels in its dynamic range. But, the motor adopted in the current study can only generate about five perceptible levels, which can be attributed to the difference in the rated rotation speed of the coin motor. The frequency of our motor only ranged from about 80 to 120 Hz. The vibrotactile TPTs have been established using variable methods on the torso.

Previous estimates of spatial vibrotactile acuities on the torso have reported 4.2 cm [44], or 5.1 cm on the back of the torso [10] by using simultaneous stimuli. Our average vibrotactile TPT at simultaneous stimulation was 6 cm, which was higher than the reported TPT ranges (5.1 cm) of the back of the torso. The variability in the vibrotactile acuities reported in these reports and our study can be attributed to experimental differences, including the frequency range of the coin motor. Notably, the TPTs of the horizontal degree appear to be more stable across the participants (see Figure 7b). Since subjects vary in waist size, measuring the spatial vibrotactile acuity of the torso as a degree is more suitable than distance. Compared to reports in the existing literature [10], the current study presents a more comprehensive investigation into the acuity of four cardinal sites of the torso in terms of spatial, intensity, and temporal parameters, not only the spatial and intensity acuities on the back of the torso.

### 4.2. Application of Current Work

The current work presents data about the vibrotactile acuities of a coin motor for its use in a vibrotactile display. In the objective test of the actuator, we not only presented the amplitude and frequency characteristics at different indentation depths but also the data of the latency shifting between two different driving voltages, which lays the foundation for precisely controlling the timing and intensity of vibration in vibrotactile displays. Additionally, this objective testing method can be implemented with other types of vibration actuators.

In the experimental study of the vibrotactile acuity of four cardinal locations of the torso, we present the data concerning spatial TPTs in horizontal and vertical directions. We also provide the data of absolute threshold levels in terms of the temporal duration and intensity, and the identifiable perceived duration and intensity levels. Additionally, measuring the horizontal TPTs in the degree in the current study enhances the applicability of the data of TPTs to torso-type vibrotactile devices. These data provide psychological guidance for designing and producing comfortable and clear perceptive vibrotactile sensations on the torso.

### 4.3. Limitations and Future Research

Although this work provides a systematic experimental investigation into the sensory characteristics of the human torso in response to a vibrotactile stimulus using coin motors, it is also subject to a number of limitations. First, extrapolating this experimental data to other body parts or types of vibrations is challenging due to the marked variability in tactile acuities across bodily skin sites. Hence, our reported results with coin motors on the torso may not be representative of other body areas. Second, regarding the eccentric rotating mass (ERM) motor used in this study, the peak vibration frequency depended on vibration amplitude and varied from 40 Hz at the detection threshold, to 150 Hz at the maximum stimulus level (Figure 1). Furthermore, two-point spatial discrimination tasks may be affected by intensity cues, as closely applied stimuli can result in the summation of intensity sensations [45].

Tactile illusions, which reveal the brain’s unconscious perceptual strategies [46,47], have been widely employed to improve the effectiveness of vibrotactile displays [4,7,48,49,50]. So far, more than 20 tactile illusions have been found [51,52]. Accordingly, the objective timing and amplitude vibrotactile acuities have strong relationships with the vibrotactile illusions in these previous findings [10]. In the future, we plan to explore tactile illusions on the torso based on the experimental findings from the current work.

## 5. Conclusions

In this paper, we designed a vibrotactile belt to psychophysically measure the vibrotactile acuities according to the three vibration parameters (intensity, temporal and spatial) of a coin motor, and objectively tested the mechanical vibration characteristics of the coin motor. The key findings at the current stage of this research are the data about the vibrotactile acuities of a coin motor in regard to its use in a vibrotactile display. In addition, the practical implications of the current research are aimed to establish foundational design principles, which provides objective and subjective data to the design of natural haptic devices with coin motors.

## Data Availability

The original contributions presented in the study are included in the article, further inquiries can be directed to the corresponding author.
